# Effect of Calcium Compound Type and Dosage on the Properties of Acid Rennet Goat’s Milk Gels

**DOI:** 10.3390/molecules26185563

**Published:** 2021-09-13

**Authors:** Małgorzata Pawlos, Agata Znamirowska, Katarzyna Szajnar

**Affiliations:** Department of Dairy Technology, Institute of Food Technology and Nutrition, College of Natural Sciences, University of Rzeszow, Ćwiklińskiej 2D, 35-601 Rzeszów, Poland; aznam@univ.rzeszow.pl (A.Z.); katarzyna.szajnar@gmail.com (K.S.)

**Keywords:** acid rennet gel, goat’s milk, calcium compounds, fortification, color, syneresis, texture, organoleptic evaluation

## Abstract

The aim of this study was to determine the effect of adding calcium compounds to processed goat’s milk, and on the properties of acid rennet goat’s milk gels, which are a middle product obtained in the manufacture of acid rennet cheese. The properties of the gels directly affect the quality of acid rennet cheeses. The analysis of raw goat’s milk was carried out, then acid rennet gels were produced with the addition of six different calcium compounds (chloride, citrate, bisglycinate, gluconate, lactate, and carbonate). The dynamics of milk fermentation were performed by monitoring the pH value of milk during acidification. The pH, syneresis, color, and texture profile were determined in the formulated acid rennet gels. An organoleptic evaluation was also performed. The study demonstrated that, not only calcium chloride, but also calcium citrate, gluconate, lactate, bisglycinate, and calcium carbonate could be used in the production of goat’s milk acid rennet gels, or the middle product in the manufacture of acid rennet curd cheese from goat’s milk. Notably, the addition of citrate, bisglycinate, and calcium carbonate in doses of 20 mg Ca 100 g^−1^ most effectively reduced syneresis compared to the control sample by 4.76% (citrate), 7.85% (bisglycinate), and 10.28% (carbonate). The hardness of the control gels ranged from 2.35 N to 2.99 N. The addition of chloride, citrate, gluconate, lactate, and calcium carbonate to the milk improved the acid rennet gel’s hardness. The addition of 20 mg Ca 100 g^−1^ as gluconate increased the hardness the most (3.61 N). When increasing the calcium dosage in the form of all compounds, there was a tendency to increase the gel’s springiness. The addition of chloride, citrate, and bisglycinate to milk did not result in a darkening of the gel’s color. The addition of calcium compounds mostly reduced the intensity of goatish taste and odor. Calcium gluconate, in particular, reduced the goatish taste the most, a taste which is not always acceptable by the consumers.

## 1. Introduction

Calcium has an essential role in milk processing and cheese manufacturing and is crucial for the proper functioning of the human body. Calcium’s primary function is as a structural component of bone and teeth, blood vessels, nails, and hair. It also participates in hormonal regulation through the activation and secretion of hormones and neurotransmitters [1,2]. However, calcium is reported to play a key role in many technological aspects of food production. Calcium suppresses deterioration, maintains integrity, and reduces cell membrane permeability in fruit [3]. Moreover, calcium ions could inactivate polygalacturonase, which is responsible for degrading cell wall materials and components such as pectin; thus, calcium plays an important role in preserving fruit quality [4]. Calcium chloride can alternatively be used in the production of surimi gel to improve its yield and higher gel strength [5].

Acid curd cheese is less rich in calcium compared to ripened cheeses due to their production technology, where 80% of the calcium may be transferred to whey. Moreover, acid curd cheese is characterized by a poorer Ca:P ratio [6]. Increasing the level of calcium in acid curd cheese is an essential aspect during its processing. It increases the content of this element in the curd [7], while also improving the curd’s compactness. The mineral compounds affect the functionality of the milk through specific and non-specific interactions with milk proteins, thus affecting the structure and stability of the proteins. Most notably, the mineral compounds influence the partitioning of caseins between the colloidal and serum phases of the milk and the ionic environment of the proteins [8]. This phenomenon could be used in the production of acid curd cheese from goat’s milk. 

As a raw material for cheese-making, goat’s milk differs from cow’s milk substantially. The lower casein content and its lower percentage of total nitrogen are the reason for the lower cheese yield. Moreover, the casein curd is delicate, not very firm, and easily loosened, which may also be the reason for its lower cheese yield. Increasing the calcium content could be achieved by modifying the technological process or enriching calcium compounds into the milk before or after the milk pasteurization process [9]. Due to the fragile structure of the acidic curd from goat’s milk and the difficulties in processing the curd, acid rennet curds are most commonly made with the addition of rennet, as the additive of rennet increases the firmness of the curd [10]. According to Tarapata et al. [11], treating casein micelles partially with rennet enhances acid coagulation and leads to the formation of an acid gel with a higher modulus of elasticity and strength. The distribution of calcium in milk affects the rate of CMP (caseinomacropeptide) releasing and the gel formation dynamics. The addition of specific calcium compounds decreases the pH of milk. The initial acidification may affect both phases of rennet coagulation, so when rennet is added to lower pH milk, gelation occurs earlier with a lower CMP cleavage. The addition of ionic calcium has a positive effect on rennet gelation. It improves interactions during aggregation by neutralizing electrostatic repulsion between casein micelles [11,12]. During acid coagulation, the acidification of milk reduces the charge of the casein micelles and causes a partial dissolving (loss) of insoluble calcium phosphate crosslinks, which alter the stability of casein micelles [13]. On the other hand, calcium plays a significant role in bridging between casein micelles in the process of rennet coagulation.

In dairy cheese making, losses of ionic calcium caused by the pasteurization of milk are usually corrected by the addition of calcium chloride [14]. A study by Gastaldi et al. [15] determined the effect of a calcium chloride addition during the combined acid and rennet coagulation of milk. It showed that the enrichment of milk with CaCl_2_ (6.25 mM) allowed for maintaining a higher degree of micelle mineralization with a pH decreasing from 6.7 to 5.10 compared to milk without calcium enrichment.

According to the EU regulations, the chemical forms of calcium that may be added to food are listed in Annex III of Commission Regulation (EC) No 1170/2009, whereas Annex II of this Regulation lists the forms of calcium that may be used in the manufacture of food supplements [16]. Calcium carrier compounds must not adversely affect the product’s color, taste, or smell, shorten its shelf life, or cause changes in the product during transport and storage. Furthermore, the fortification treatment must be inexpensive enough to increase the price of the final product substantially. Moreover, the mineral carriers used must be entirely safe for human health [17].

Since the color of a product shapes consumers’ perceptions, investigating the effects of color on the human senses is both important and interesting. According to Chudy et al. [18], the white color of milk results from its physical and chemical structure. The natural color of milk occurs from light reflection by dispersed fat globules, calcium caseinate, and calcium phosphate. Furthermore, milk naturally contains two classes of pigments: water-soluble and fat-soluble. Caprine milk contains no β-carotene, only retinol, and xanthophylls. Goats convert β-carotene into vitamin A, which does not have a color [18,19]. Calcium compounds may change the taste, color, and appearance of dairy products. Palacios et al. [20] reported that most calcium compounds are white or colorless, and therefore do not change the final product’s color. However, some insoluble calcium salts may lighten the color of the food products. In contrast, soluble calcium salts could interact with other food components, such as tannins, causing darkening. Furthermore, calcium can interact with anthocyanins containing secondary hydroxyl groups, causing a color change from red to blue [20].

Previous studies investigating the effect of adding various calcium compounds have been conducted on the properties of cow’s fermented milk, whereas there are few studies on calcium-enriched goat’s milk acid and acid rennet gels.

The aim of this study was to determine the effect of the type of calcium compound addition to processing goat’s milk and its dose on the properties of acid rennet goat’s milk gels, which are an indirect form obtained in the manufacture of acid rennet cheese.

## 2. Results and Discussion

### 2.1. Quality of Goat’s Milk

The chemical composition of raw goat’s milk depends on various factors: genetic, environmental (nutrition and production season), and physiological, such as the lactation stage, animal health condition, or udder health [21,22]. In our study, milk was obtained from hybrid goats without signs of udder infection during the autumn season.

The composition and physicochemical characteristic of raw goat’s milk is presented in Table 1. This composition is comparable to the results of other studies.

Currò et al. [23] reported that the total protein content of goat milk varies from 2.10 to 5.61 g 100 g^−1^, and the fat content from 1.90 to 8.10 g 100 g^−1^. According to Pandya and Ghodke [24], and Raynal-Ljutovac et al. [19], caprine milk’s average total protein content was 3.52 g 100 g^−1^, which is 0.8 g 100 g^−1^ more compared to our study. A study by Currò et al. [23] indicated that goat milk protein content is influenced by the goat breed, lactation stage, and animal feeding. The relatively low protein content of milk found in this study might be related to the low amount of protein in forage from the Podkarpackie grasslands, grown under poorer climatic conditions and in poor soil classes [25]. Furthermore, milk fat content is a variable quantity and quality component of milk, determined by the lactation stage, season, breed, genotype, and nutrition [19]. Barłowska et al. [21] compared the chemical composition of goat milk from conventional and organic farms. Organic milk was characterized by a significantly lower total protein and casein content in comparison with milk from conventional farms. These authors claimed that the differences shown between seasons in terms of the chemical composition of milk from goats housed on organic farms are related, besides nutrition, to their stage of lactation. According to Barłowska et al. [26], organic goat milk from the summer season showed a protein content of 2.78 g 100 g^−1^, which is comparable to the results of our study.

It should be emphasized that the processing suitability of goat milk is related to the chemical composition of the milk, as low protein and fat content may influence the yield in cheese production and its properties [27,28]. However, lactose content, a carbon source for microorganisms, determines the intensity of lactic fermentation and the quality of fermented products [29,30]. Pastuszka et al. [31] studied the chemical composition of milk of goats of different breeds and showed lactose content ranging from 4.02 (Alpine breed) to 4.97 g 100 g^−1^ (Somali breed). A more comprehensive range of lactose content (from 4.08 to 5.09 g 100 g^−1^) in goat milk was reported by Barlowska et al. [32]. The lactose content found in goat milk in our study was within this range.

Table 1 shows the results indicating the density and freezing point of goat milk. These parameters are potential indicators of milk water dilution and may influence the quality of the milk gel. The density of processed milk was 1.028 g mL^−1^. In the study of Strzałkowska et al. [33], goat milk was characterized by a density ranging from 1.026 to 1.029 g mL^−^^1^, depending on the stage of lactation. Similarly, Park et al. [34] conducted density measurements on goat’s milk, obtaining results ranging from 1.029 to 1.039 g mL^−1^. Moreover, the authors determined the minimum freezing point of goat’s milk to be −0.540 °C, and the maximum at −0.573 °C. A higher freezing point value (−0.533 to −0.550 °C) was reported by Mayer and Fiechter [35]. In our study, the density and freezing point of goat milk was in accordance with results obtained by Strzałkowska et al. [33], Park et al. [34] and Mayer and Fiechter [35].

The results in Table 1 show the pH value of goat milk, which was 6.68. Similar results were obtained by Mayer and Fiechter [35], who obtained a pH value in goat milk in the range of 6.49 to 6.67. Another study by Park et al. [34] determined a pH value in a close range (6.50–6.80).

In the European Union, regulations allow a maximum of 1.5 million CFU mL^−1^ in goat milk. The TBC (total bacterial count) showed in Table 1 is much lower, which indicates that the evaluated milk meets the criteria of Regulation 1662/2006 [36]. Znamirowska et al. [37] determined a lower bacterial cell count (120,500 CFU mL^−1^) in goat milk. Another study reported TBC in goat milk of 359,000 CFU mL^−1^ [38]. Sikora and Kawęcka [39], investigated the suitability for cheese making using goat milk from the Saanen breed, and reported a total bacterial count of 100,000 CFU mL^−1^.

Barłowska et al. [21] determined the SCC (somatic cell count) in goat milk at the level of 5.82 log cells mL^−1^. According to Litwińczuk et al. [40], 1 million somatic cells in 1 mL (6.00 log cells mL^−1^) of goat milk can be considered a physiologically acceptable value. The SCC results in Table 1 exceed this limit, which is probably related to the autumn season for milk production. Danków et al. [41] found that the season of production significantly affects the SCC in goat milk, as the lowest level of the SCC was determined in spring (5.69 log cells mL^−1^) and the highest in autumn (6.24 log cells mL^−1^). A similar trend was observed by Brodziak et al. [42], finding the decreased cytological quality of milk during the autumn-winter season. This was likely related to the stage of lactation because, at the end of the lactation period with a decrease in milk yield, there occurs an increase in the number of somatic cells.

### 2.2. Effect of Calcium Dose and Calcium Compound on the pH Value of Goat’s Milk after Pasteurization

The application of various calcium compounds in doses from 0 mg to 20 mg of calcium for every 100 g of goat milk was used to determine the possibility of their use in the production of acid rennet goat’s milk gels as alternatives to calcium chloride. Calcium compounds show significant differences in their practical application in dairy technology. According to Ziarno et al. [7], the most significant negative aspect of using these compounds was the appearance of calcium ionic forms, which significantly increased the susceptibility of casein micelles to aggregation and, consequently, protein precipitation. As a result, the heat treatment process of goat milk may be complicated.

In our study, after pasteurization, control goat milk (0 mg Ca 100 g^−1^ milk) showed the same pH value (6.57) in all groups (Table 2). However, depending on the calcium compound added to the milk, the pH values changed significantly in the acidic or alkaline direction. The addition of chloride, gluconate, and lactate in a dose of 20 mg Ca 100 g^−1^ milk resulted in a significant decrease in the pH value of milk compared to the control (*p* ≤ 0.05). Chloride (20 mg Ca 100 g^−1^) acidified the milk the most, decreasing the pH value by 0.21. In contrast, increasing doses of bisglycinate to the milk increased the pH value. The ability of bisglycinate to neutralize milk could be used to increase enrichment doses with compounds that give milk an acidic character. Using the appropriate proportions between the alkaline-forming bisglycinate and calcium compounds that increase the acidity of milk may result in a desirable increase in the amount of the introduced mineral [4,38]. Moreover, the observation of cooled fortified milk samples carried out in this study did not show coagulation resulting in curdling of milk proteins.

Calcium compounds have different chemical structures and solubility in milk. Ziarno et al. [43] showed that water-soluble calcium compounds (lactate, gluconate, lactogluconate, and chloride) caused a decrease in pH value and an increase in titratable acidity of cream. Similarly, in our study, these calcium compounds increased the acidity of goat milk. In a study carried out by Znamirowska et al. [44], the enrichment of cow’s milk with 100 mg Ca 100 g^−1^ in the form of lactate decreased the pH value by up to 0.67 units compared to control milk. In our study, the addition of calcium lactate (20 mg Ca 100 g^−1^) decreased the pH value of goat milk by 0.09.

An essential characteristic of poorly water-soluble compounds (carbonate and citrate) is their neutrality to the milk protein, even at increased temperature, so they can be added to milk before pasteurization without concern for reducing the thermal stability of proteins and their precipitation [7]. Goat milk with the addition of 20 mg Ca 100 g^−1^ in the form of citrate and carbonate after pasteurization showed pH values in the range of 6.58–6.62 (Table 2).

### 2.3. The Dynamics of Acidification of Goat’s Milk with the Addition of Calcium Compounds

**Figure 1:**
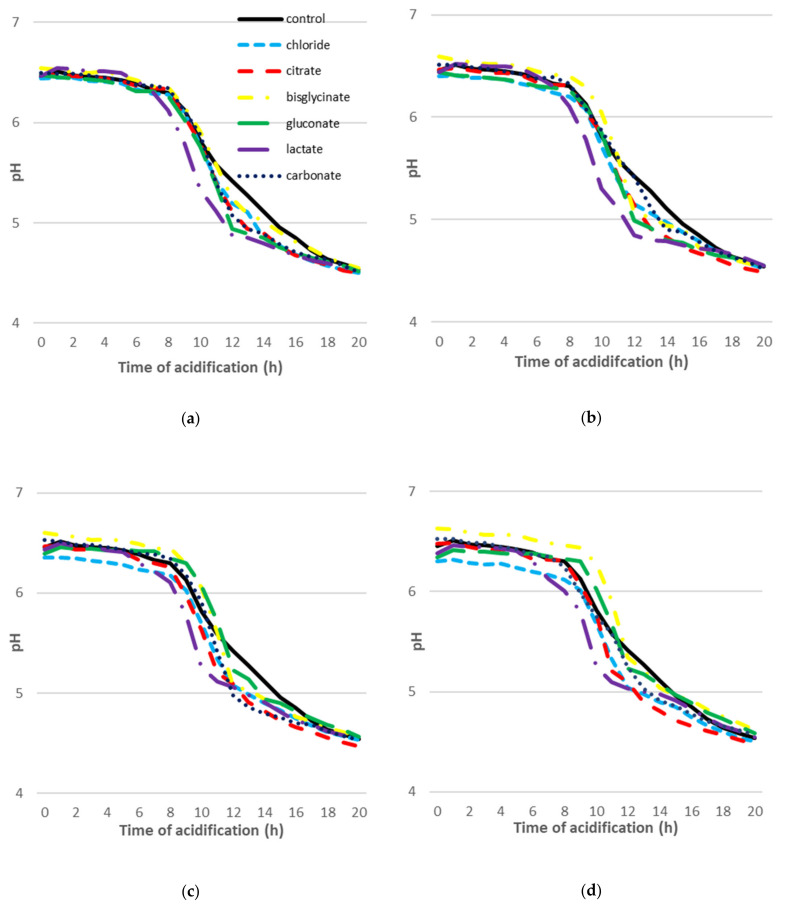
Acidification process of control goat milk samples (without calcium addition) and with addition: 5 mg Ca 100 g^−1^ of milk (**a**), 10 mg Ca 100 g^−1^ of milk (**b**), 15 mg Ca 100 g^−1^ of milk (**c**), 20 mg Ca 100 g^−1^ of milk (**d**), in the form of calcium chloride, calcium citrate, calcium bisglycinate, calcium gluconate, calcium lactate, and calcium carbonate.

Figure 1 shows changes in pH during the acidification process of goat milk with the addition of six calcium compounds, in doses: 5 mg Ca 100 g^−1^ of milk (a), 10 mg Ca 100 g^−1^ of milk (b), 15 mg Ca 100 g^−1^ of milk (c), and 20 mg Ca 100 g^−1^ of milk (d).

In all milk samples with the addition of calcium compounds, and in the control sample, fermentation with mesophilic strains was carried out until a pH value of 4.6 (±0.5) was reached and a gel was obtained. The pH value of goat milk without the calcium addition at the beginning of incubation was 6.56–6.57. In the control sample, a pH value of 4.64 was reached after 18 h of incubation. The addition of 5 mg Ca 100 g^−1^ to goat milk in the form of chloride, citrate, gluconate, and lactate resulted in the formation of a gel after 17 h of incubation, with a pH in the range of 4.62–4.65 (Figure 1a). However, the pH decreased most rapidly in milk with the addition of calcium chloride. The acidification process was the longest in the milk sample with the addition of calcium in the form of bisglycinate (19 h; pH = 4.6). An increase of calcium dose to 10 mg Ca 100 g^−1^ of milk caused the prolongation of the incubation time up to 19 h only in goat milk with calcium addition in the form of lactate (Figure 1b). The application of calcium bisglycinate at a dose of 20 mg Ca 100 g^−1^ milk increased the incubation time the most, up to 20 h (pH = 4.62).

After 12 h of incubation, there was a notable decrease in the pH value of all samples, regardless of the level of calcium addition, compared to the control (Figure 1a–d). Meanwhile, all samples with calcium bisglycinate (5–20 mg Ca 100 g^−1^ milk) showed a higher pH value for 12 h of incubation than those without the calcium addition. The addition of calcium citrate at doses of 5, 10, 15, and 20 mg Ca 100 g^−1^ milk resulted in the highest reduction in the milk’s pH value after 19 h of incubation.

In fermented milk production, the pH of the product is typically lowered to 4.0–4.8, compared to milk’s natural pH of 6.6–6.8. Along with lowering the milk pH, the various acid-basic groups of the milk components (organic and inorganic phosphate, citrate, and carboxyl acid residues) become increasingly protonated, which has two main effects. Primarily, the neutralization of the charge of the κ-casein segments protruding from the surface of casein micelles leads to a decrease in solubility and thus a reduction in steric and electrostatic stability concomitant aggregation of casein micelles [45,46,47]. Furthermore, colloidal calcium phosphate (CCP), which was initially present in the casein micelle, becomes solubilized, resulting in a change in the internal structure of the micelle. The degree of CCP solubilization depends on pH and temperature [46,47].

### 2.4. The Effect of Calcium Addition on the Properties of Acid Rennet Goat’s Milk Gels

Gelation is a general method of converting liquids into solids and has been used since ancient times to produce various food products with specific textures. Foods such as gelatin-based desserts are the simplest food gels, consisting of a water-gelatin gel with added sweetener, flavor, and color. Agar, starch, pectin, iota- and kappa-carrageenans, alginates, and gellan gum can form gels. In this gelation method, hydrogen bonds allow numerous water molecules to join the polymer molecule, building a double helix structure of the molecule and aggregating polymer molecules. An example of polysaccharide-based gelatin is kappa-carrageenan, a sulphated polysaccharide extracted from different species of red seaweed. This polysaccharide forms a thermo-reversible gel, and its gelation is influenced by the temperature, concentration, type, and amount of metal salts, as well as the presence of food ingredients, such as sugars [48,49]. In a study by Yang et al. [50], the sol-gel transition, gel network strength, and stability of k-carrageenan were greatly improved upon the addition of sucrose. For some polysaccharides, aside from hydrogen bonds, the presence of mono- and divalent ions, such as calcium, is crucial. Calcium ions enhance the double helix formation and induce a strong aggregation of the resulting pairs of polysaccharide molecules, leading to a solid viscoelastic structure [51].

Differently, all cheeses begin as a simple gel are then further transformed to remove certain ingredients and add others. In contrast to polysaccharide-based gelation, this milk-protein-based gelation is an irreversible process [52]. Calcium is commonly used in cheese making because its addition to milk increases the concentration of ionic calcium in milk, reduces rennet-induced milk clotting time, and increases calcium retention in cheeses. Moreover, it may increase dry matter content and protein and fat retention [11]. In our study, the gel was formed by acid rennet coagulation, with lactic fermentation bacteria producing lactic acid to provide better conditions for the activity of the rennet.

The physical and structural properties of fermented milk gels are influenced by process parameters, such as temperature, time, and mechanical factors (stirring, pumping, and aeration), and by changes in the structure of milk proteins during the pre- and post-fermentation processes. Moreover, each factor could contribute to reduced curd stability in fermented milk [53]. The specific properties of goat milk form the different texture and rheological properties of fermented milk compared to fermented cow milk [54,55,56]. According to Znamirowska et al. [37], some mineral compounds added to milk before pasteurization can improve the textural properties of milk acid gels.

#### 2.4.1. The pH Value of Goat’s Milk Gels

The production of acid curd cheese is based on the process of casein coagulation, which occurs in milk as a result of directed fermentation under the influence of lactic fermentation bacteria. The lactic acid produced during fermentation affects the reduction of the pH value to the isoelectric point of casein (pH = 4.60), and the formation of a casein gel with an ordered structure in the spaces in which water is enclosed along with the components dissolved in it [29]. According to Tarapata et al. [11], the technology of producing acid rennet curd is based on adding a starter culture and a relatively small amount of rennet to the processing milk. Under these conditions, the milk is slowly acidified, and gel formation occurs when the pH is lowered near the isoelectric point of casein.

Acid rennet gel without calcium addition showed a pH value in the range of 4.61–4.63. With increasing doses of calcium in the form of calcium chloride, gluconate, and lactate, the pH value of the gels decreased (Table 3).

The addition of calcium chloride and gluconate to goat’s milk at increasing doses resulted in a significant decrease in the gel’s pH value (*p* ≤ 0.05) compared to the control sample. An opposite correlation was found in goat gel with citrate, bisglycinate, and carbonate. The increasing dose of calcium resulted in the formation of acid and rennet gel with a significantly higher pH value (*p* ≤ 0.05) than in the control sample. The performed analysis of variance ANOVA (Table 4) indicated that the pH value of the milk gels was significantly affected by the calcium compound and the interaction of calcium compound and calcium dose.

In Ziarno and Więcławski [55] study, after 24 h of incubation with mesophilic microflora, the pH value of media with the addition of calcium lactate was higher by about 0.2 units compared to media without the addition of this calcium compound. Ziarno et al. [7] evaluated buttermilk produced from cow’s milk with the addition of 72 mg% calcium in the form of citrate, which showed a pH value of 4.57, and with the addition of 26 mg% calcium in the form of lactate, with a pH value of 4.62. According to these authors’ experiments, the addition of calcium compounds to cow’s milk resulted in a decrease in the pH value of buttermilk by 0.05 to 0.1 pH units compared with buttermilk without added compounds.

#### 2.4.2. Syneresis of Goat’s Milk Gels

Syneresis is considered among the most visible and notable defects in fermented milk resulting from the accumulation of whey on the surface of milk gels [37]. The phenomenon of syneresis occurs due to the shrinkage of the gel, which leads to the separation of whey. The level of syneresis may be related to the higher calcium content of goat milk compared to cow milk, and the occurrence of ionic interactions between casein micelles in the network formed by proteins. The process of whey separation may also be related to the rigidity and stability of the protein network and other factors, such as protein denaturation, low pH, high total acidity, and the type and intensity of heat treatment [37,56,57]. Moschopoulou et al. [58] reported that higher fat levels in goat milk also increase the water holding capacity of milk gels. However, Dmytrów [59] showed that whey separation was significantly affected by the type of starter culture used. The author concluded that mesophilic bacteria used in acid curd inoculants, due to different acidification of the medium, caused shrinkage of the casein curd and thus determine the differences in whey volume secreted. The results of Mulawka et al.’s [29] study showed that changes in the colloidal system of curd and increasing acidity of the medium affecting the water absorption of milk proteins might determine the whey release process.

Acid rennet gel without a calcium addition was characterized by whey leakage of 28.05% to 32.27%. In acid rennet gels with a calcium addition in the form of chloride, gluconate, and lactate, the decreased pH value in both milk and acid rennet gels was observed, and a more intensive syneresis than in control milk was found. In contrast, syneresis was reduced by adding citrate, bisglycinate, and calcium carbonate (Table 5). The highest syneresis (38.03%) was determined in acid rennet gel with the addition of 20 mg Ca 100 g^−1^ (in the form of gluconate), and it was significantly higher in comparison with the control (*p* ≤ 0.05). An increase in syneresis was observed with an increasing calcium addition. However, a decrease in syneresis with an increasing calcium dose was found in milk gels with calcium bisglycinate.

At lower a pH, the gel was harder, most probably because the bonds in the protein network were stronger. Increased gel acidification may have resulted in stronger gel shrinkage and more intense syneresis. In addition, the course of the fermentation dynamics (slow or fast) affected the formation of the protein matrix, the ability of the gel to retain components, and the intensity of whey separation. The excessively fast fermentation rate of goat’s milk could lead to a stronger aggregation of casein micelles while lowering the water content, inducing micelle dehydration, forming granular curd structures, and intense whey separation, which is not reabsorbed by the acid gel of the milk [7,11]. In Liu et al.’s [60] study, it was indicated that high renneting (about 55 and 74% and low pH (4.8–5.0) contributed to high levels of spontaneously separated whey (50–70%), which means that highly acidified milk forms a less stable gel network to entrap serum.

Two-way ANOVA analysis of variance (Table 4) indicated that compound and calcium dose significantly affected the level of syneresis. The combined effect of the alcium dose and calcium compound was also significant.

#### 2.4.3. Color of Goat’s Milk Gels

Color is one of the first attributes perceived by the senses that consumers use to evaluate the quality of food products [61]. This study was determined to test if the application of different calcium compounds at different dosages would cause significant changes in the color of goat’s milk acid rennet gels. Table 6 shows the effect of different calcium compounds on the color parameters L*, a*, b*, C, h°.

The addition of calcium chloride at a dose of 5–20 mg Ca 100 g^−1^ milk did not significantly affect the lightness (L*) of color compared to the control. In comparison to the control sample, gels with an addition of calcium citrate in the dose of 5–20 mg Ca 100 g^−1^ of milk and calcium bisglycinate in the doses of 10, 15, and 20 mg Ca 100 g^−1^ of milk were characterized by a lighter color. However, color darkening was caused by the addition of calcium in the form of gluconate (doses of 5–20 mg Ca 100 g^−1^), lactate (doses of 5–20 mg Ca 100 g^−1^), and carbonate (doses of 5–20 mg Ca 100 g^−1^) (Table 5). Mineral salts change the distribution of caseins between colloidal milk and phase serum and the ionic medium of proteins. Minerals can modify the matrix structure of casein micelles in acidic curds by causing the color of protein curds to turn gray [62].

The two-way ANOVA indicated that the brightness L* was significantly affected by the calcium compound, calcium dose, and the interaction of these two factors (Table 4).

In goat’s milk acid rennet gels with calcium chloride and calcium carbonate, regardless of the dose used, the intensity of the green color a* decreased compared to the measurements of the a* parameter for the control sample. However, the addition of citrate, bisglycinate, gluconate, and lactate did not affect the changes in the color parameter a* (Table 5).

Regardless of the calcium dosage used, the calcium chloride addition decreased the yellow intensity of the acid rennet gel compared to the control sample. In contrast, the yellow intensity (b*) increased in the calcium gluconate sample at all dosages used. The presence of calcium citrate, calcium bisglycinate, calcium lactate, and calcium carbonate introduced in the milk did not significantly affect the b* color parameter compared to the control sample (Table 5).

Hue (h°) determines the dominant wavelength in the spectrum of that color. This wavelength determines the hue of the color [63]. In our study, in most samples the application of calcium compounds did not change the h° parameter, only the addition of calcium lactate increased the h° parameter compared to the control (Table 5).

Chroma (C) describes the clarity of a color (how much the dominant wavelength is “polluted” by others). Colors whose spectrum contains a narrow range of wavelengths are perceived as more saturated. Colors with low saturation are called pastel colors [63]. The results of color saturation (C) indicate that these color coordinates are dependent on all tested factors (calcium compound and calcium dose) and the interaction between calcium compound and calcium dose (Table 4).

#### 2.4.4. Texture of Goat’s Milk Gels

Texture describes the physical properties of a product, such as hardness, adhesiveness, viscosity, and springiness. All these characteristics are structural elements and can be perceived by the human senses. Consequently, texture is a basic determinant of the quality of fermented dairy products [64]. Fermented goat’s milk is characterized by a weaker texture and lower apparent viscosity, and a higher tendency to syneresis, than fermented cow’s milk [65]. The weaker consistency and lower viscosity of fermented goat milk products is due to their delicate microstructure, which is less resistant and more sensitive to rapid deformation [66]. Therefore, goat milk acid gels are less dense and softer compared to cow milk acid gel. These properties are directly related to the smaller diameter of casein micelles, the lower degree of hydration, the lower mineralization and casein content of milk, especially αs1 casein, and the smaller diameter of non-protein nitrogen in goat milk relative to cow milk [34]. The rheological and textural properties of fermented milk are affected by acid aggregation of casein micelles and exopolysaccharide (EPS) production by starter cultures during incubation. Moreover, the rheological properties and texture of fermented milk are influenced by the amount and structure of EPS released and the interactions occurring between EPS and casein micelles [37,65,67]. Simultaneously, these characteristics are influenced by the chemical composition of the milk (mainly the moisture content), the heat treatment of the milk, and the starter cultures used. According to Donato and Guyomarc’h [68], the heat treatment of milk promotes the aggregation process, which affects the formation of stronger milk gels. Furthermore, it reduces the degree of acidification necessary to form a combined protein matrix in fermented milk. The aggregation of whey proteins and casein is essential for the physical and chemical properties of casein micelles, and therefore creates the texture of fermented products directly. Siemianowski et al. [69] consider that acid curd texture parameters are essential in acid curd cheese technology, as they influence the process of its preparation, which includes cutting, stirring and heating for drying.

Control gel (without calcium addition) was characterized by a hardness from 2.35 N to 2.99 N (Table 7). The addition of chloride, citrate, gluconate, lactate, and calcium carbonate increased the gel hardness. However, the addition of calcium bisglycinate to goat’s milk decreased the hardness of the acid rennet gel (*p* ≤ 0.05). The ANOVA analysis of variance (Table 4) indicates that the type of calcium compound significantly influenced the hardness of the milk gels. Ziarno and Więcławski [55] observed the weakening of curd firmness and stability formed during the incubation of 100 mL of milk containing 4 g of calcium lactate. The analysis conducted by Znamirowska et al. [44] on the texture components of calcium-enriched milk beverages showed that the beverages were characterized by a significantly lower hardness and springiness values than the control beverages.

The cohesiveness of the control goat gel, which determines the strength of the internal bonds constituting the gel structure, showed values ranging from 0.19 to 0.43 (Table 7). The addition of 20 mg of Ca (for 100 g of goat’s milk) resulted in the cohesiveness of the acid rennet gels from 0.16 to 0.71 (Table 7).

Depending on the type of calcium compound used, a decrease or increase in gel cohesiveness was observed. The addition of calcium chloride, bisglycinate, gluconate, lactate, and carbonate increased gel cohesiveness. In contrast, increasing the dose of calcium citrate to milk caused a decrease in gel cohesiveness. A significant increase in cohesiveness values in gels was obtained by adding calcium chloride and calcium lactate (*p* ≤ 0.05). In the study by Znamirowska et al. [44], the cohesiveness of milk beverages fermented using *Bifidobacterium* showed higher values in fermented milk fortified with calcium and magnesium lactates than in controls.

A two-way ANOVA analysis of variance (Table 4) indicated that the cohesiveness of the acid rennet gels made from goat milk was significantly affected by the type of calcium compound. The interaction between the type of calcium compound and calcium dose was also significant in the cohesiveness determination.

Springiness was considered as another texture component of the gels analyzed, which determined the distance over which the deformed sample would regain its dimension after the deforming forces were removed (Table 7). The springiness of the analyzed control gel (0 mg Ca 100 g^−1^) showed values ranging from 6.47 mm to 14.69 mm. With an increased calcium dose added to milk, a tendency to increase the springiness of gels with all types of calcium compounds was observed. The lowest springiness was found in the gel with 20 mg Ca (for 100 g of milk) in the form of calcium citrate (7.74 mm), while the highest (16.95 mm) in the gel with calcium gluconate. Acid rennet gels with 20 mg Ca 100 g^−1^ in the form of chloride, citrate, gluconate and lactate were characterized by a significantly higher springiness than the control (*p* ≤ 0.05). ANOVA analysis of variance (Table 4) confirms that the springiness of the gels was significantly affected by the type of calcium compound and the interaction between the compound and calcium dose.

The level of attraction to a surface is referred to as adhesion or adhesiveness. The lower the numerical value of the product’s adhesion, the lower the adhesiveness. In sensory terms, adhesiveness can be defined as the degree of adherence of a chewed mass to the palate. It is also a term characterizing the force required to remove food from the oral surface during eating [70,71].

Control gels (0 mg Ca 100 g^−1^) showed an adhesiveness ranging from 0.02 mJ to 0.23 mJ (Table 7). The addition of chloride, citrate, bisglycinate, lactate, and calcium carbonate decreased the adhesiveness of goat acid rennet gels. However, the highest adhesiveness of acid rennet gel of the tested calcium compounds was caused by adding calcium carbonate to milk. The ANOVA analysis of variance (Table 4) confirms that the adhesiveness of the gels was significantly affected by the type of calcium compound.

Pawlos et al. [72] reported that the addition of calcium in the form of bisglycinate reduced the adhesiveness of kefirs made from cow’s milk on 21 days of refrigerated storage. The authors showed an adhesiveness of 4.10 mJ in control kefirs. Kefirs made from milk with an addition of 30 mg Ca 100 g^−1^ showed an adhesiveness lower by 1.83 mJ than the control. Domagała [64] showed that the lowest adhesiveness characterized yoghurts made from goat’s milk compared to yoghurts made from cow’s and sheep’s milk. The author reports that such properties of goat’s milk yoghurts are due to lower levels of dry matter and total protein in goat’s milk, compared to the content of these components in cow’s and sheep’s milk. Ziarno and Zaręba [73] found that the adhesion of cow’s milk yoghurts decreased with increasing storage time.

#### 2.4.5. The Organoleptic Evaluation of Goat’s Milk Gels

The consumer acceptance of goat dairy products is low due to its goatish flavor resulting from high levels of caproic, caprylic, and capric fatty acids. According to various authors [74,75], the off-taste and odor found in goat products could also be the result of the high-temperature processing of goat milk. Park et al. [34] and Znamirowska et al. [37] studies showed that the addition of mineral compounds reduced the intensity of goat and sour taste and odor in goat milk products.

An organoleptic evaluation confirmed that adding some calcium compounds to goat milk influenced the organoleptic quality of the acid rennet gels (Table 8). The major descriptors for lowering the organoleptic evaluation notes were texture, sour and goatish taste, and fermentation odor.

The addition of calcium chloride to goat milk significantly affected the sour taste and fermentation odor (*p* ≤ 0.05), resulting in a decrease in the intensity of these properties in the gels compared to the control sample. The addition of calcium citrate and gluconate significantly (*p* ≤ 0.05) influenced the sour taste and fermentation odor of goat acid rennet gels compared to the control. Gels with the addition of 10 and 20 mg Ca 100 g^−1^ milk were characterized by a better, more compact consistency, and less noticeable goatish taste. In contrast, the addition of calcium bisglycinate reduced the consistency of the gels, compared to the control. The consistency of these gels was less compact and more spreadable. Acid rennet gels made from goat milk, with the addition of all calcium compounds, were characterized by a similar milky-creamy and salty taste compared to control gels. Mostly, the addition of lactate and carbonate to the milk did not change the organoleptic characteristics of the gels. However, it was observed that the introduction of these compounds into goat’s milk significantly (*p* ≤ 0.05) reduced the intensity of the fermentation odor of the gels. It should be pointed that the addition of six calcium compounds did not increase the intensity of off-taste and off-odor in the gels, which, with the reduction of the goatish taste, is a positive aspect in the production of goat milk dairy products.

The analysis of variance ANOVA indicated that the applied calcium dose significantly influenced the organoleptic characteristics of the analyzed acid rennet gels from caprine milk.

Sheehan et al. [76] reported that the odor and taste described as an animal (goatish) or waxy is one of the most dominant characteristics of goat milk products. Moreover, the soft aroma of fermented goat milk products is explained by the lower content of volatile aroma compounds (mainly diacetyl) and carbon dioxide formed during fermentation with mesophilic cultures. Szwocer et al. [77] found that goat milk has a lower citrate content, and thus a more deficient composition of flavorings in fermented products.

## 3. Materials and Methods

### 3.1. Materials

Raw morning and chilled (4 °C) bulk goat’s milk for producing acid rennet gels was collected in September directly from an organic farm in the Podkarpacie region (Zabratówka, Poland) from different colored goats of mixed breeds. Before the analyses and acid rennet gels production, milk was filtrated to remove dirt and foreign particles.

IBCm Bacto Kit 500 and IBCm SCC Kit reagents were purchased from Bentley Instruments Inc., (Chaska, MN, USA). Calcium chloride (CaCl_2_ × 6H_2_O), calcium lactate (C_6_H_10_CaO_6_ × 5H_2_O), and calcium carbonate (CaCO_3_) were purchased from Chempur (Piekary Śląskie, Polska). Calcium citrate (Ca_3_(C_6_H_5_O_7_)_2_ × 4H_2_O) was purchased from Gadot Biochemical Industries Ltd. (Haifa, Israel), and calcium bisglycinate (C_4_H_4_CaN_2_O_4_) was purchased from Olimp Laboratories (Dębica, Poland), whereas calcium gluconate (C_12_H_22_CaO_14_ × H_2_O) was purchased from Sigma Aldrich (Saint Luis, MO, USA). The starter culture of mesophilic lactic fermentation bacteria G500 (*Lactococcus lactis* subsp. *lactis*, *Lactococcus lactis* subsp. *cremoris*, *Lactococcus lactis* subsp. *lactis* biovar *diacetylactis*) was purchased from CSK Food Enrichment (Wageningen, The Netherlands), and Beaugel 5 rennet was purchased from Coquard (Villefranche sur Saône, France).

All of the reagents used were of analytical reagent grade.

### 3.2. Raw Goat’s Milk Analysis

The total bacterial count (TBC) and somatic cell count (SCC) were determined in the Bacto Count IBC M/SCC semi-automatic counter (Bentley Instruments Inc., Chaska, MN, USA). The chemical composition (protein, fat, lactose, and total solids) and freezing point were determined in milk and the milk products composition analyzer, Bentley B-150 (Bentley Instruments Inc., Chaska, MN, USA). The density of goat milk was performed at a temperature of 20 °C, according to Raţu et al. [78]. The pH value was determined using a digital pH meter Toledo FiveEasy TM (Mettler Toledo, Greifensee, Switzerland) using an electrode InLab^®®^Solids Pro-ISM (Mettler Toledo, Switzerland).

### 3.3. Acid Rennet Gels Production

The goat’s milk was divided into thirty batches, each with a different amount of calcium and calcium compound added: milk with calcium chloride addition (0, 5, 10, 15, 20 mg Ca 100 g^−1^ of milk); milk with calcium citrate addition (0, 5, 10, 15, 20 mg Ca 100 g^−1^ of milk); milk with calcium bisglycinate addition (0, 5, 10, 15, 20 mg Ca 100 g^−1^ of milk); milk with calcium gluconate addition (0, 5, 10, 15, 20 mg Ca 100 g^−1^ of milk); milk with calcium lactate addition (0, 5, 10, 15, 20 mg Ca 100 g^−1^ of milk); and milk with calcium carbonate addition (0, 5, 10, 15, 20 mg Ca 100 g^−1^ of milk). The calcium dose was calculated on the molecular weight of the calcium compound used. Control milks and milks with calcium addition were pasteurized (90 °C, 5 min). After the heat treatment, milk was cooled to 28 °C and each batch of milk was inoculated with 0.20% (*w*/*w*) starter culture of mesophilic lactic fermentation bacteria 15 min prior to addition of 0.02% (*v*/*w*) of rennet. The rennet strength was 1/3000 with 150 mg of active chymosin per liter. Then the milk was gently mixed and incubated at 28 °C, until the pH reached 4.6 (±0.50) value.

### 3.4. The Dynamics of Milk Acidification

The dynamics of goat’s milk acidification was determined by recording pH changes during the coagulation process with modifications, according to Tarapata et al. [11]. The pH value was measured with a digital pH meter Toledo FiveEasy TM (Mettler Toledo, Greifensee, Switzerland) using an electrode InLab^®®^Solids Pro-ISM (Mettler Toledo, Greifensee, Switzerland), every 1 h until pH reached the value 4.6 (±0.5).

### 3.5. pH of Acid Rennet Gel

The pH determination in the formulated acid rennet gels was performed with a pH-meter (FiveEasy Mettler Toledo, Greifensee, Switzerland) using an electrode InLab^®®^Solids Pro-ISM (Mettler Toledo, Greifensee, Switzerland).

### 3.6. Syneresis (%) of Acid Rennet Gels

Syneresis was estimated according to the method proposed by Szajnar et al. [79], using Laboratory 110 Refrigerated Centrifuge LMC-4200R (Biosan SIA, Riga, Latvia). Ten grams of gel was transferred into 50 mL plastic tube and centrifuged at 3160× *g* for 10 min, 5 °C. The syneresis was estimated as the released whey over the original weight.

### 3.7. Color of Acid Rennet Gels

The color was analyzed with a colorimeter (the Precision Colorimeter, Model NR 145, Shenzhen, China) using the CIELAB system. The image brightness was determined with the parameter L* and chromaticity using a*, b*, C, h°. Before testing, the device was calibrated on a white and black reference standard [79].

### 3.8. Texture Analysis of Acid Rennet Gels

The TPA test determined the texture profile using a CT3 Texture Analyzer (Brookfield AMETEK, Middleboro, MA, USA) with Texture Pro CT (Brookfield AMETEK, Middleboro, MA, USA) software. The sample dimensions were cylinder 66 mm × 33.86 mm, and the temperature of the sample was 8 °C. The experiment was conducted using the acrylic probe TA 3/100 with the following settings: distance 15 mm, contact load 0.1 N, and measurement speed 1 mm/s [79].

### 3.9. Qualitative Organoleptic Evaluation of Acid Rennet Gels

The organoleptic properties of the acid rennet gels were evaluated on a 9 cm linear scale, non-structured. The following descriptors were studied: consistency, milky-creamy taste, salty taste, sour taste, goatish taste, off-taste, fermentation odor, goatish odor, and off-odor. The trained team assessed the organoleptic parameters: ten women and ten men (age 20–40), chosen from academic staff and students. Gels samples (~50 mL) were served in random order, in transparent plastic containers, and coded with three-digit numbers. The samples of gels were evaluated on a 9-point scale, with markings at both ends. The left end denoted the least intense, the least characteristic feature: weak, liquid consistency, impalpable milky-creamy taste, salty taste, sour taste, goatish taste, off-taste, fermentation odor, goatish odor, and off-odor. The right end denoted the most characteristic feature: compact consistency, the most intense milky-creamy taste, salty taste, sour taste, goatish taste, off-taste, fermentation odor, goatish odor, and off-odor [37,80,81].

### 3.10. Statistical Analysis

Statistical analysis was performed according to Szajnar et al. [79]. From the obtained results, the mean and standard deviation were worked out statistically in the software Statistica 13.1 (StatSoft, Tulsa, OK, USA). A two-way ANOVA was used to investigate the overall effect of calcium compound×calcium dose on acid rennet gel properties. The significance of differences between the averages was estimated with Tukey’s test (*p* ≤ 0.05). The experiment was repeated three times on different occasions. Three samples were tested for each acid rennet gel variant, and it was repeated for three trials.

## 4. Conclusions

The experiment showed that the applied calcium compounds in doses 0–20 mg Ca 100 g^−1^ of milk did not decrease the thermal stability of goat milk proteins and allowed for pasteurization at 90 °C/15 s, which indicates their safe application in goat milk processing. Moreover, the conducted studies indicate that not only calcium chloride but also calcium citrate, gluconate, lactate, bisglycinate, and calcium carbonate are suitable for the production of acid rennet gels, an intermediate form in goat’s milk acid rennet curd cheese production. Notably, the addition of citrate, bisglycinate, and calcium carbonate was most effective in reducing syneresis. Our study showed that the texture of acid rennet gel could be enhanced by using the appropriate calcium compound at the correct dosage, as the addition of chloride, citrate, gluconate, lactate and calcium carbonate to milk increased the hardness of acid rennet gel. Increasing the dose of calcium in the form of all compounds allowed a tendency to increase the gel’s springiness. It should also be noted that adding some calcium compounds (chloride, citrate, or bisglycinate) to milk did not cause a darkening of the color of the acid rennet gels. The addition of calcium compounds in general reduced the intensity of the goatish taste and odor; in particular calcium, gluconate reduced the goatish taste the most. The obtained results have an applicative character and may have an essential role in developing new functional goat’s milk dairy products.

## Figures and Tables

**Table 1:** Composition and physicochemical characteristic of raw goat’s milk.

Properties	Mean ± SD ^1^
Protein, g 100 g^−1^	2.71 ± 0.31
Fat, g 100 g^−1^	2.79 ± 0.49
Lactose, g 100 g^−1^	4.70 ± 0.24
Total solids, g 100 g^−1^	10.40 ± 0.73
Density, g mL^−1^	1.028 ± 0.01
Freezing point, °C	−0.573 ± 0.020
pH	6.68 ± 0.08
TBC ^2^, log CFU mL^−1^	5.63 ± 0.06
SCC ^3^, log cells mL^−1^	6.01 ± 0.05

^1^ SD—standard deviation; ^2^ TBC—total bacterial count; ^3^ SCC—somatic cell count.

**Table 2:** The pH value of goat’s milk after pasteurization.

Calcium Compound	Calcium Dose, mg Ca 100 g^−1^ of Milk				
	0	5	10	15	20
Chloride	6.57e ± 0.01	6.53d ± 0.01	6.49c ± 0.01	6.42b ± 0.01	6.36a ± 0.01
Citrate	6.57a ± 0.02	6.57a ± 0.01	6.57a ± 0.01	6.58a ± 0.01	6.58a ± 0.01
Bisglycinate	6.57a ± 0.01	6.67b ± 0.01	6.68b ± 0.01	6.71c ± 0.01	6.73c ± 0.01
Gluconate	6.57c ± 0.01	6.57c ± 0.01	6.53b ± 0.02	6.51b ± 0.01	6.46a ± 0.01
Lactate	6.57c ± 0.01	6.57c ± 0.01	6.55bc ± 0.01	6.53b ± 0.01	6.48a ± 0.01
Carbonate	6.57a ± 0.01	6.60b ± 0.01	6.62b ± 0.01	6.63b ± 0.02	6.62b ± 0.01

Mean ± standard deviation; a–e Means with different letters in rows indicate statistically significant differences at *p* < 0.05; 9 samples × 30 batches = 270 samples.

**Table 3:** pH of acid rennet goat’s milk gels.

Calcium Compound	Calcium Dose, mg Ca 100 g^−1^ of Milk				
	0	5	10	15	20
Chloride	4.63b ± 0.01	4.60a ± 0.01	4.60a ± 0.01	4.59a ± 0.01	4.59a ± 0.01
Citrate	4.61a ± 0.02	4.63b ± 0.01	4.64b ± 0.01	4.65c ± 0.01	4.65c ± 0.01
Bisglycinate	4.62a ± 0.01	4.63ab ± 0.01	4.63ab ± 0.01	4.64b ± 0.01	4.64b ± 0.01
Gluconate	4.63b ± 0.01	4.63b ± 0.00	4.63b ± 0.01	4.61ab ± 0.01	4.59a ± 0.01
Lactate	4.63a ± 0.01	4.64a ± 0.00	4.62a ± 0.01	4.62a ± 0.02	4.62a ± 0.01
Carbonate	4.62a ± 0.02	4.66b ± 0.00	4.67c ± 0.00	4.69d ± 0.01	4.70d ± 0.00

Mean ± standard deviation; a–d Means with different letters in rows indicate statistically significant differences at *p* < 0.05; 9 samples × 30 batches = 270 acid rennet goat milk gels samples.

**Table 4:** Analysis of variance (ANOVA) *p* values on the effects of calcium compound and calcium dose on pH, color parameters: L*, a*, b*, C, h°, syneresis, hardness, cohesiveness, springiness, adhesiveness, consistency, milky-creamy taste, salty taste, sour taste, goatish taste, off-taste, fermentation odor, goatish odor and off-odor of acid rennet goat’s milk gels.

Properties		Calcium Compound *p* Values	Calcium Dose *p* Values	Calcium Compound × Calcium Dose *p* Values
pH		↑ 0.0000	ns 0.6258	↑ 0.0000
**Color**	L*	↑ 0.0000	↑ 0.0183	↑ 0.0000
	a*	↑ 0.0000	ns 0.1101	↑ 0.0000
	b*	↑ 0.0000	ns 0.2207	↑ 0.0000
	C	↑ 0.0000	↑ 0.0000	↑ 0.0000
	h°	↑ 0.0000	ns 0.7426	↑ 0.0000
Syneresis		↑ 0.0000	↑ 0.0165	↑ 0.0000
Hardness		↑ 0.0000	ns 0.5999	ns 0.3173
Cohesiveness		↑ 0.0164	ns 0.0927	↑ 0.0000
Springiness		↑ 0.0000	ns 0.1509	↑ 0.0480
Adhesiveness		↑ 0.0000	ns 0.7660	ns 0.5509
Consistency		ns 0.5890	↑ 0.0000	ns 0.9713
Milky-creamy taste		ns 0.7675	↑ 0.0000	ns 0.5449
Salty taste		ns 0.9283	↑ 0.0000	ns 0.9362
Sour taste		ns 0.5309	↑ 0.0000	ns 0.3527
Goatish taste		ns 0.0523	↑ 0.0081	ns 0.9691
Off-taste		ns 0.7274	↑ 0.0000	ns 0.2153
Fermentation odor		ns 0.8597	↑ 0.0000	ns 0.9983
Goatish odor		ns 0.9995	↑ 0.0007	ns 0.9999
Off-odor		ns 0.9998	↑ 0.0000	ns 0.3556

Calcium compound×calcium dose = interaction; ↑ indicates significant effect *p* ≤ 0.05; ns: no significant effect.

**Table 5:** Syneresis (%) of acid rennet goat’s milk gels.

Calcium Compound	Calcium Dose, mg Ca 100 g^−1^ of Milk				
	0	5	10	15	20
Chloride	28.14a ± 2.73	30.37a ± 1.27	30.04a ± 2.32	31.05a ± 0.86	34.09b ± 0.70
Citrate	29.66b ± 1.72	26.16ab ± 0.31	26.81ab ± 0.51	24.92a ± 0.74	24.90a ± 0.12
Bisglycinate	29.63c ± 2.43	26.90bc ± 3.55	23.59b ± 1.57	22.50ab ± 3.56	21.78a ± 2.31
Gluconate	31.63a ± 0.21	31.08ab ± 0.95	34.16ab ± 3.35	37.69b ± 1.04	38.03b ± 2.06
Lactate	28.05a ± 2.70	30.76ab ± 1.05	30.50ab ± 1.14	31.70b ± 2.41	34.73b ± 2.11
Carbonate	32.27b ± 1.87	31.04b ± 2.13	22.40a ± 2.93	21.88a ± 1.45	21.99a ± 0.83

Mean ± standard deviation; a–c Means with different letters in rows indicate statistically significant differences at *p* < 0.05; 9 samples × 30 batches = 270 acid rennet goat milk gels samples.

**Table 6:** Color of acid rennet goat’s milk gels.

Calcium Compound	Color	Calcium Dose, mg Ca 100 g^−1^ of Milk				
		0	5	10	15	20
Chloride	L*	92.04a ± 0.27	92.07a ± 0.05	92.36a ± 0.40	92.34a ± 0.30	92.33a ± 0.04
	a*	−1.51c ± 0.05	−1.47b ± 0.01	−1.47b ± 0.04	−1.48b ± 0.06	−1.42a ± 0.01
	b*	7.00c ± 0.06	6.89b ± 0.05	6.89b ± 0.14	6.83a ± 0.09	6.87b ± 0.03
	C	7.17b ± 0.07	6.95a ± 0.05	6.95a ± 0.14	6.99a ± 0.08	7.00a ± 0.03
	h°	102.21b ± 0.39	102.44b ± 0.12	102.46b ± 0.50	102.44b ± 0.62	101.44a ± 0.09
Citrate	L*	90.44a ± 0.25	90.86ab ± 0.53	90.82b ± 0.33	90.71b ± 0.19	90.87b ± 0.37
	a*	−1.50a ± 0.05	−1.51a ± 0.02	−1.53a ± 0.04	−1.55a ± 0.03	−1.55a ± 0.03
	b*	7.00a ± 0.06	7.04a ± 0.05	7.05a ± 0.09	7.00a ± 0.09	6.99a ± 0.12
	C	7.07a ± 0.07	7.31c ± 0.05	7.50d ± 0.08	7.18b ± 0.08	7.00a ± 0.07
	h°	101.12a ± 0.39	101.26a ± 0.22	101.71a ± 0.36	101.76a ± 0.36	101.89a ± 0.41
Bisglycinate	L*	90.42ab ± 0.27	90.39a ± 0.03	90.65b ± 0.06	90.72b ± 0.14	91.72c ± 0.12
	a*	−1.52a ± 0.05	−1.56a ± 0.01	−1.55a ± 0.00	−1.56a ± 0.03	−1.55a ± 0.04
	b*	7.01a ± 0.06	7.01a ± 0.04	7.06a ± 0.04	7.00a ± 0.05	6.98a ± 0.19
	C	6.97a ± 0.07	7.06b ± 0.04	7.02ab ± 0.04	7.08b ± 0.06	6.99ab ± 0.22
	h°	101.21a ± 0.39	101.39a ± 0.08	101.77b ± 0.05	101.96bc ± 0.45	102.17c ± 0.35
Gluconate	L*	92.14d ± 0.27	91.62c ± 0.04	91.39b ± 0.06	91.22b ± 0.08	90.92a ± 0.14
	a*	−1.50a ± 0.05	−1.48a ± 0.02	−1.49a ± 0.01	−1.51a ± 0.02	−1.52a ± 0.02
	b*	6.99a ± 0.06	7.09ab ± 0.02	7.11b ± 0.04	7.22c ± 0.05	7.23c ± 0.05
	C	6.97a ± 0.07	7.09ab ± 0.02	7.19b ± 0.10	7.17b ± 0.05	7.17b ± 0.05
	h°	100.21a ± 0.39	100.93a ± 0.12	100.92a ± 1.40	100.90a ± 0.05	100.88a ± 0.12
Lactate	L*	92.02b ± 0.27	90.03a ± 0.27	90.08a ± 0.06	90.03a ± 0.03	90.03a ± 0.04
	a*	−1.51a ± 0.05	−1.55a ± 0.01	−1.55a ± 0.00	−1.54a ± 0.01	−1.56a ± 0.01
	b*	7.00a ± 0.06	6.98a ± 0.07	6.97a ± 0.03	7.02a ± 0.05	7.01a ± 0.04
	C	7.17b ± 0.07	7.05a ± 0.07	7.05a ± 0.03	7.13b ± 0.01	7.12b ± 0.03
	h°	102.21a ± 0.39	102.74b ± 0.14	102.79b ± 0.07	102.82b ± 0.15	102.83b ± 0.03
Carbonate	L*	92.06d ± 0.27	91.04c ± 0.21	90.84b ± 0.01	90.67a ± 0.08	90.53a ± 0.26
	a*	−1.51b ± 0.05	−1.48b ± 0.03	−1.43a ± 0.01	−1.43a ± 0.01	−1.44ab ± 0.10
	b*	7.00a ± 0.06	7.01a ± 0.03	6.99a ± 0.05	6.99a ± 0.08	7.00a ± 0.27
	C	7.17a ± 0.07	7.15a ± 0.03	7.11a ± 0.01	7.11a ± 0.08	7.11a ± 0.28
	h°	102.21b ± 0.39	102.27b ± 0.16	102.10b ± 0.07	101.95a ± 0.07	101.82a ± 0.28

Mean ± standard deviation; a–d Means with different letters in rows indicate statistically significant differences at *p* < 0.05; 9 samples × 30 batches = 270 acid rennet goat milk gels samples.

**Table 7:** Texture parameters of acid rennet goat’s milk gels.

Properties	Calcium Compound	Calcium Dose, mg Ca 100 g^−1^ of Milk				
		0	5	10	15	20
Hardness, N	Chloride	2.99a ± 0.05	3.15a ± 0.35	3.37a ± 0.24	2.90a ± 0.28	3.22a ± 0.39
	Citrate	2.65a ± 0.04	2.79ab ± 0.09	3.19b ± 0.17	2.83ab ± 0.45	2.80ab ± 0.22
	Bisglycinate	2.35b ± 0.30	1.97ab ± 0.45	1.67ab ± 0.32	1.41a ± 0.64	1.53ab ± 0.81
	Gluconate	2.65a ± 0.04	2.88a ± 0.09	3.18b ± 0.18	3.38b ± 0.33	3.61b ± 0.01
	Lactate	2.99a ± 0.05	2.71a ± 0.44	3.55a ± 0.58	3.37a ± 0.51	3.06a ± 0.14
	Carbonate	2.65a ± 0.04	2.72b ± 0.01	2.84b ± 0.47	2.28a ± 0.05	3.02a ± 0.31
Cohesiveness	Chloride	0.25a ± 0.01	0.37b ± 0.02	0.37b ± 0.01	0.36b ± 0.03	0.40b ± 0.01
	Citrate	0.19a ± 0.17	0.37b ± 0.03	0.16a ± 0.14	0.42c ± 0.05	0.16a ± 0.14
	Bisglycinate	0.43a ± 0.08	0.49a ± 0.01	0.51a ± 0.09	0.45a ± 0.06	0.53a ± 0.05
	Gluconate	0.29a ± 0.10	0.26a ± 0.09	0.47b ± 0.03	0.43b ± 0.03	0.34ab ± 0.01
	Lactate	0.25a ± 0.01	0.33a ± 0.00	0.76b ± 0.00	0.71b ± 0.06	0.71b ± 0.03
	Carbonate	0.26a ± 0.11	0.25a ± 0.09	0.36a ± 0.05	0.26a ± 0.16	0.37a ± 0.05
Springiness, mm	Chloride	6.47a ± 0.10	7.66ab ± 1.06	8.14b ± 0.14	8.34b ± 0.10	8.68b ± 0.15
	Citrate	6.75a ± 0.08	6.80a ± 0.55	7.17ab ± 0.51	7.54b ± 0.47	7.74b ± 0.41
	Bisglycinate	14.69a ± 0.63	14.51a ± 0.41	14.51a ± 0.47	14.87a ± 0.46	14.90a ± 0.47
	Gluconate	14.42a ± 0.58	14.43a ± 0.89	15.25ab ± 0.79	15.74ab ± 1.01	16.95b ± 0.40
	Lactate	6.47a ± 0.10	8.17b ± 0.29	8.65bc ± 1.43	9.67c ± 0.38	14.89d ± 0.08
	Carbonate	8.75a ± 0.09	8.93a ± 0.05	9.26a ± 0.56	8.72a ± 0.89	8.76a ± 0.28
Adhesiveness, mJ	Chloride	0.15a ± 0.09	0.12a ± 0.03	0.10a ± 0.01	0.10a ± 0.01	0.11a ± 0.01
	Citrate	0.17b ± 0.05	0.08a ± 0.03	0.17b ± 0.09	0.12ab ± 0.04	0.08a ± 0.03
	Bisglycinate	0.10a ± 0.08	0.14a ± 0.13	0.10a ± 0.10	0.14a ± 0.08	0.07a ± 0.08
	Gluconate	0.02a ± 0.03	0.02a ± 0.03	0.02a ± 0.00	0.05a ± 0.05	0.03a ± 0.00
	Lactate	0.15b ± 0.09	0.07ab ± 0.06	0.00a ± 0.00	0.00a ± 0.00	0.10b ± 0.00
	Carbonate	0.23a ± 0.06	0.23a ± 0.06	0.24a ± 0.07	0.21a ± 0.01	0.21a ± 0.02

Mean ± standard deviation; a–d Means with different letters in rows indicate statistically significant differences at *p* < 0.05; 9 samples × 30 batches = 270 acid rennet goat milk gels samples.

**Table 8:** Organoleptic evaluation of acid rennet goat’s milk gels.

Calcium Compound	Properties	Calcium Dose, mg Ca 100 g^−1^ of Milk		
		0	10	20
Chloride	Consistency	2.83a ± 0.40	2.83a ± 0.94	3.67a ± 0.81
	Milky-creamy taste	3.00a ± 1.54	3.67a ± 0.82	2.83a ± 1.33
	Salty taste	1.83a ± 0.34	2.00a ± 0.55	2.50a ± 0.52
	Sour taste	5.33b ± 0.31	3.83a ± 0.83	4.33ab ± 0.23
	Goatish taste	5.67a ± 1.87	3.50a ± 0.64	4.17a ± 0.83
	Off-taste	1.00a ± 0.00	1.00a ± 0.00	1.17a ± 0.11
	Fermentation odor	4.83b ± 0.66	3.50ab ± 1.05	2.50a ± 0.84
	Goatish odor	2.25a ± 0.91	1.67a ± 0.21	1.83a ± 0.33
	Off-odor	1.00a ± 0.00	1.00a ± 0.00	1.00a ± 0.00
Citrate	Consistency	2.83a ± 0.40	3.33ab ± 0.63	4.17b ± 0.23
	Milky-creamy taste	3.00a ± 0.54	3.67a ± 0.43	3.00a ± 0.79
	Salty taste	1.83a ± 0.34	2.33a ± 0.21	1.83a ± 0.28
	Sour taste	5.33b ± 0.31	3.67a ± 0.27	3.83a ± 0.58
	Goatish taste	5.67b ± 0.87	3.67a ± 0.37	3.67a ± 0.63
	Off-taste	1.00a ± 0.00	1.00a ± 0.00	1.17a ± 0.11
	Fermentation odor	4.83c ± 0.66	3.50b ± 0.55	2.33a ± 0.52
	Goatish odor	2.25a ± 0.91	1.67a ± 0.21	1.67a ± 0.23
	Off-odor	1.00a ± 0.00	1.00a ± 0.00	1.00a ± 0.00
Bisglycinate	Consistency	2.83b ± 0.40	1.83a ± 0.58	1.67a ± 0.21
	Milky-creamy taste	3.00a ± 1.54	3.00a ± 0.53	2.67a ± 0.75
	Salty taste	1.83a ± 0.34	1.83a ± 0.17	2.00a ± 0.26
	Sour taste	5.33b ± 0.31	4.00a ± 0.63	3.67a ± 0.63
	Goatish taste	5.67b ± 0.87	4.33a ± 0.63	3.67a ± 0.75
	Off-taste	1.00a ± 0.00	1.00a ± 0.00	1.17a ± 0.41
	Fermentation odor	4.83c ± 0.66	3.50b ± 0.35	1.83a ± 0.37
	Goatish odor	2.25a ± 0.51	1.67a ± 0.21	1.67a ± 0.23
	Off-odor	1.00a ± 0.00	1.00a ± 0.00	1.00a ± 0.00
Gluconate	Consistency	2.83a ± 0.40	3.67b ± 0.34	4.67c ± 0.34
	Milky-creamy taste	3.00a ± 1.54	2.83a ± 1.37	2.67a ± 1.37
	Salty taste	1.83a ± 0.34	2.07a ± 0.60	2.00a ± 0.26
	Sour taste	5.33b ± 0.31	3.83a ± 0.72	3.83a ± 0.98
	Goatish taste	5.67b ± 0.87	3.17a ± 0.94	3.00a ± 0.60
	Off-taste	1.00a ± 0.00	1.00a ± 0.00	1.00a ± 0.11
	Fermentation odor	4.83c ± 0.66	3.17b ± 0.17	1.67a ± 0.82
	Goatish odor	2.25a ± 0.91	1.67a ± 0.21	1.67a ± 0.33
	Off-odor	1.00a ± 0.00	1.00a ± 0.00	1.00a ± 0.00
Lactate	Consistency	2.83a ± 0.40	3.00a ± 0.41	3.17a ± 0.47
	Milky-creamy taste	3.00a ± 0.54	3.00a ± 0.89	3.17a ± 0.14
	Salty taste	1.83a ± 0.34	1.83a ± 0.38	1.50a ± 0.55
	Sour taste	5.33b ± 0.31	3.83a ± 0.47	3.50a ± 0.64
	Goatish taste	5.67b ± 0.87	3.50a ± 0.64	3.67a ± 0.21
	Off-taste	1.00a ± 0.00	1.00a ± 0.00	1.00a ± 0.00
	Fermentation odor	4.83c ± 0.66	3.50b ± 0.25	2.33a ± 0.51
	Goatish odor	2.25b ± 0.31	1.67ab ± 0,21	1.23a ± 0.28
	Off-odor	1.00a ± 0.00	1.00a ± 0.00	1.00a ± 0.00
Carbonate	Consistency	2.83a ± 0.40	2.87a ± 0.82	3.33a ± 0.75
	Milky-creamy taste	3.00a ± 0.54	2.93a ± 0.37	3.00a ± 0.10
	Salty taste	1.83a ± 0.34	1.77a ± 0.73	1.83a ± 0.28
	Sour taste	5.33a ± 0.31	5.83a ± 0.48	4.80a ± 0.84
	Goatish taste	5.67a ± 0.87	4.17a ± 0.79	4.17a ± 0.98
	Off-taste	1.00a ± 0.00	1.00a ± 0.00	1.17a ± 0.11
	Fermentation odor	4.83c ± 0.66	3.33b ± 0.21	1.83a ± 0.17
	Goatish odor	2.25a ± 0.91	1.87a ± 0.21	1.83a ± 0.33
	Off-odor	1.00a ± 0.00	1.00a ± 0.00	1.00a ± 0.00

Mean ± standard deviation; a–c Means with different letters in rows indicate statistically significant differences at *p* < 0.05; *n* = 60.

## References

[1] Kanahara M., Kai H., Okamura T., Wada T., Suda K., Imaizumi T., Sagawa K. (2008). Usefulness of high-concentration calcium chloride solution for correction of activated partial thromboplastin time (APTT) in patients with high-hematocrit value. Thromb. Res..

[2] Szeleszczuk Ł., Kuras M. (2014). Znaczenie wapnia w metabolizmie człowieka i czynniki wpływające na jego biodostępność w diecie. The role of calcium in human metabolism and factors affecting its bioavailability. Biul. Wydziału Farm. WUM.

[3] Aguayo E., Requejo-Jackman C., Stanley R., Woolf A. (2015). Hot water treatment in combination with calcium ascorbate dips increases bioactive compounds and helps to maintain fresh-cut apple quality. Postharvest Biol. Technol..

[4] Zhang L., Wang P., Sun X., Chen F., Lai S., Yang H. (2020). Calcium permeation property and firmness change of cherry tomatoes under ultrasound combined with calcium lactate treatment. Ultrason. Sonochemistry.

[5] Zhou Y., Yang H. (2019). Effects of calcium ion on gel properties and gelation of tilapia (*Oreochromisniloticus*) protein isolates processed with pH shift method. Food Chem..

[6] Siemianowski K., Szpendowski J. (2012). Możliwość zwiększania zawartości wapnia w serach twarogowych w świetle dotychczasowych badań. Possibilities of tvarog cheeses enrichment with calcium in the light of hitherto existing research. Nauk. Inżynierskie I Technol..

[7] Ziarno M., Zaręba D., Piskorz J. (2009). Wzbogacanie maślanki w wapń, magnez oraz białka serwatkowe. Fortifying buttermilk with calcium, magnesium, and whey proteins. Żywność Nauka Technol. Jakość.

[8] Augustin M.A., Clarke P.T. (1990). Effects of added salts on the heat stability of recombined concentrated milk. J. Dairy Res..

[9] Szpendowski J., Kłobukowski J., Prokop E. (2005). Wpływ dodatku chlorku wapnia i ogrzewania mleka na skład chemiczny serów twarogowych. The effect of calcium chloride added to milk and milk heating on the chemical composition of cottage cheeses. Żywność Nauka Technol. Jakość.

[10] Danków R., Pikul J. (2011). Przydatność technologiczna mleka koziego do przetwórstwa. Technological suitability of goat milk for processing. Nauka Przyr. Technol..

[11] Tarapata J., Smoczyński M., Maciejczyk M., Żulewska J. (2020). Effect of calcium chloride addition on properties of acid-rennet gels. Int. Dairy J..

[12] Sørensen I., Le T.T., Larsen L.B., Wiking L. (2019). Rennet coagulation and calcium distribution of raw milk reverse osmosis retentate. Int. Dairy J..

[13] Lucey J.A., McSweeney P.L.H., O’Mahony J.A. (2016). Acid coagulation of milk. Advanced Dairy Chemistry.

[14] Jaworski J., Kuncewicz A., Ziajka S. (2007). Właściwości fizykochemiczne mleka. Physicochemical properties of milk. Mleczarstwo.

[15] Gastaldi E., Pellegrini A., Lagaude B., Tarodo de la Fuente B. (1994). Functions of Added Calcium in Acid Milk Coagulation. J. Food Sci..

[16] (2009). Commission Regulation (EC) No 1170/2009 of 30 November 2009 amending Directive 2002/46/EC of the European Parliament and of Council and Regulation (EC) No 1925/2006 of the European Parliament and of the Council as regards the lists of vitamin and minerals and their forms that can be added to foods, including food supplements (Text with EEA relevance). Off. J. Eur. Union.

[17] Kowalska M., Ambroziak A., Aljewicz M., Cichosz G. (2012). Wzbogacone w wapń i magnez produkty mleczarskie. Fortification of dairy products by calcium and magnesium. Postępy Tech. Przetwórstwa Spożywczego.

[18] Chudy S., Bilska A., Kowalski R., Teichert J. (2020). Colour of milk and milk products in CIE L*a*b* space. Med. Weter..

[19] Raynal-Ljutovac K., Lagriffoul G., Paccard P., Guillet I., Chilliard Y. (2008). Composition of goat and sheep milk products: An update. Small Rumin. Res..

[20] Palacios C., Cormick G., Hofmeyr G.J., Garcia-Casal M.N., Peña-Rosas J.P., Betrán A.P. (2020). Calcium-fortified foods in public health programs: Considerations for implementation. Ann. N. Y. Acad. Sci..

[21] Barłowska J., Wolanciuk A., Kędzierska-Matysek M., Litwińczuk Z. (2013). Wpływ sezonu produkcji na podstawowy skład chemiczny oraz zawartość makro- i mikroelementów w mleku krowim i kozim. Effect of production season on basic chemical composition and content of macro- and microelements in cow’s and goat’s milk. Żywność Nauka Technol. Jakość.

[22] Kędzierska-Matysek M., Barłowska J., Litwińczuk Z., Koperska N. (2015). Content of macro- and microelements in goat milk in relation to the lactation stage and region of production. J. Elem..

[23] Currò S., De Marchi M., Claps S., Salzano A., De Palo P., Manuelian C.L., Neglia G. (2019). Differences in the Detailed Milk Mineral Composition of Italian Local and Saanen Goat Breeds. Animals.

[24] Pandya A.J., Ghodke K.M. (2007). Goat and sheep products rather than cheeses and yoghurts. Small Rumin. Res..

[25] Barszczewski J. (2015). Stan trwałych użytków zielonych i ich wykorzystanie w kraju. Status of permanent grasslands and their use domestically. Woda-Środowisko-Obsz. Wiej. Rozpr. Nauk. I Monogr..

[26] Barłowska J., Litwińczuk Z., Domaradzki P., Pastuszka R., Wójcik-Saganek A. (2016). Wpływ sezonu na skład chemiczny i profil kwasów tłuszczowych mleka krowiego i koziego produkowanego w gospodarstwach ekologicznych. Impact of season on chemical composition and fatty acid profile of cow’s and goat’s milk produced in organic farms. Żywność Nauka Technol. Jakość.

[27] Barłowska M., Litwińczuk Z., Wolanciuk A., Pastuszka R. (2014). Skład chemiczny, jakość cytologiczna i przydatność technologiczna mleka krów trzech ras o umaszczeniu czerwono-białym żywionych systemem TMR. The chemical composition, cytological quality and technological suitability of the milk of three breeds of red and white cows fed in a TMR system. Rocz. Nauk. Pol. Tow. Zootech..

[28] Król J., Brodziak A., Kędzierska-Matysek M., Brodziak A., Zaborska A., Litwińczuk A. (2018). The effect of selected factors on yield and protein and mineral retention in traditionally produced tvarog. J. Elem..

[29] Mulawka E., Dmytrów I., Mituniewicz-Małek A., Godula K. (2019). Rodzaj kultury starterowej a wybrane cechy fizykochemiczne sera twarogowego w czasie przechowywania. Type of starter culture and selected physicochemical characteristics of curd cheese (tvarog) during storage. Żywność Nauka Technol. Jakość.

[30] Żylińska J., Siemianowski K., Bohdziewicz K., Pawlikowska K., Kołakowski P., Szpendowski J., Bardowski J. (2014). Kultury starterowe do produkcji twarogów kwasowych—Rola i oczekiwania. Postępy Mikrobiol..

[31] Pastuszka R., Barłowska J., Litwińczuk Z. (2015). Walory odżywcze i prozdrowotne mleka koziego. Nutritional value and health-promoting properties of goat milk. Med. Weter..

[32] Barłowska J., Szwajkowska M., Litwińczuk Z., Król J. (2011). Nutritional Value and Technological Suitability of Milk from Various Animal Species Used for Dairy Production. Compr. Rev. Food Sci. Food Saf..

[33] Strzałkowska N., Jóźwik A., Bagnicka E., Krzyżewski J., Horbańczuk K., Pyzel B., Horbańczuk J.O. (2009). Chemical composition, physical traits and fatty acid profile of goat milk as related to the stage of lactation. Anim. Sci. Pap. Rep..

[34] Park Y.W., Haenlein G.F.W., Hui Y.H. (2007). Goat milk, its products and nutrition. Handbook of Food Products Manufacturing.

[35] Mayer H., Fiechter G. (2012). Physical and chemical characteristics of sheep and goat milk in Austria. Int. Dairy J..

[36] (2006). Commission Regulation (EC) No 1662/2006 of 6 November 2006 amending Regulation (EC) No 853/2004 of the European Parliament and of the Council laying down specific hygiene rules for food of animal origin (Text with EEA relevance). Off. J. Eur. Union.

[37] Znamirowska A., Szajnar K., Pawlos M. (2019). Organic magnesium salts fortification in fermented goat’s milk. Int. J. Food Prop..

[38] Znamirowska A., Kalicka D., Pawlos M., Szajnar K. (2015). Quality of yoghurts from goat’s milk enriched with magnesium chloride. J. Microbiol. Biotechnol. Food Sci..

[39] Sikora J., Kawęcka A. (2015). Jakość produktu tradycyjnego z mleka koziego—Sera podkarpackiego białego. Quality of the white podkarpacki cheese, a traditional goat milk product. Wiadomości Zootech..

[40] Litwińczuk A., Kędzierska-Matysek M., Barłowska J. (2007). Wydajność i jakość mleka kóz o różnych genotypach alfa-s1-kazeiny z rejonu Wielkopolski i Podkarpacia. Productivity and quality of milk from goats of different alpha-s1-casein genotypes from Wielkopolska and Podkarpacie regions. Med. Weter..

[41] Danków R., Cais-Sokolińska D., Pikul J., Wójtowski J. (2003). Jakość cytologiczna mleka koziego. Cytological quality of goat’s milk. Med. Weter..

[42] Brodziak A., Król J., Barłowska J., Litwińczuk Z. (2014). Effect of production season on protein fraction content in milk of various breeds of goats in Poland. Int. J. Dairy Technol..

[43] Ziarno M., Semeniuk E., Kycia K. (2004). Wpływ dodatku soli wapnia na stabilność mleka przeznaczonego do produkcji sera typu cottage cheese. The impact of the calcium salts addition on the stability of milk used in the cottage cheese production. Żywność Nauka Technol. Jakość.

[44] Znamirowska A., Buniowska M., Kuźniar P. (2018). Wzbogacanie mleczanem magnezu i wapnia mlecznych napojów fermentowanych przez *Bifidobacterium animalis* ssp. Lactis Bb-12. Fortification of fermented milk beverages by Bifidobacterium animalis ssp. lactis bb-12 with magnesium and calcium lactate. Zesz. Probl. Postępów Nauk Rol..

[45] Robles-Rodríguez C.E., Szymańska E., Huppertz T., Özkan L. (2021). Dynamic modeling of milk acidification: An empirical approach. Food Bioprod. Process..

[46] Li X.Y., Cheng M., Li J., Zhao X., Qin Y.S., Chen D., Wang J.M., Wang C.F. (2020). Change in the structural and functional properties of goat milk protein due to pH and heat. J. Dairy Sci..

[47] Lucey J.A. (2017). Formation, structural properties, and rheology of acid-coagulated milk gels. Cheese: Chemistry, Physics and Microbiology, Volume 1.

[48] Azizi R., Farahnaky A. (2016). Ultrasound assisted-viscosifying of kappa carrageenan without heating. Food Hydrocoll..

[49] Stenner R., Matubayasi N., Shimizu S. (2016). Gelation of carrageenan: Effects of sugars and polyols. Food Hydrocoll..

[50] Yang Z., Yang H., Yang H. (2018). Effects of sucrose addition on the rheology and microstructure of k-carrageenan gel. Food Hydrocoll..

[51] Sow L.C., Toh N.Z.Y., Wong C.W., Yang H. (2019). Combination of sodium alginate with tilapia fish gelatin for improved texture properties and nanostructure modification. Food Hydrocoll..

[52] Van der Linden E., Foegeding E.A., Kasapis S., Norton I.T., Ubbink J.B. (2009). Gelation: Principles, Models and Applications to Proteins, Chapter 2. Modern Biopolymer Science.

[53] Lucey J.A. (2004). Cultured dairy products: An overview of their gelation and texture properties. Int. J. Dairy Technol..

[54] Delgado K.F., Da Silva Frasao B., Costa M.P. (2017). Different Alternatives to Improve Rheological and Textural Characteristics of Fermented Goat Products—A Review. Rheol. Open Access.

[55] Ziarno M., Więcławski S. (2006). Wpływ dodatku mleczanu wapnia na rozwój bakterii fermentacji mlekowej w bulionie MRS i w mleku. The Influence of the Calcium Lactate Addition on the Growth of the Lactic Acid Bacteria in the MRS Broth and Milk. Żywność Nauka Technol. Jakość.

[56] Jacob M., Jaros D., Rohm H. (2011). Recent advances in milk clotting enzymes. Int. J. Dairy Technol..

[57] Stelios K., Emmanuel A. (2004). Characteristics of set type yoghurt made from caprine or ovine milk and mixtures of the two. Int. J. Food Sci. Technol..

[58] Moschopoulou E., Sakkas L., Zoidou E., Theodorou G., Sgouridou E., Kalathaki C., Moatsou G., Chatzigeorgiou A., Politis I., Moatsou G. (2018). Effect of Milk Kind and Storage on the Biochemical, Textural and Biofunctional Characteristics of Set-Type Yoghurt. Int. Dairy J..

[59] Dmytrów I. (2015). Wpływ probiotycznych bakterii kwasu mlekowego na stabilność przechowalniczą kwasowych serów twarogowych. Effect of lactic acid probiotic bacteria on storage stability of acid curd cheeses (tvarog). Żywność Nauka Technol. Jakość.

[60] Liu X.T., Zhang H., Wang F., Luo J., Guo H.Y., Ren F.Z. (2014). Rheological and structural properties of differently acidified and renneted milk gels. J. Dairy Sci..

[61] Giusti M.M., Wrolstad R.E. (2003). Acylated Anthocyanins from Edible Sources and Their Applications in Food Systems. Biochem. Eng. J..

[62] Augustin M.A. (2000). Mineral Salts and Their Effect on Milk Functionality. Aust. J. Dairy Technol..

[63] Rożnowski J. (2006). Ocena barwy produktów spożywczych. Evaluation of food color. Laboratorium.

[64] Domagała J. (2009). Instrumental Texture, Syneresis and Microstructure of Yoghurts Prepared from Goat, Cow and Sheep Milk. Int. J. Food Prop..

[65] Domagała J., Wszołek M. (2008). Wpływ sposobu zagęszczania oraz rodzaju szczepionki na teksturę i podatność na synerezę jogurtu i biojogurtów z mleka koziego. Effect of concentration method and starter culture type on the texture and susceptibility to syneresis of yoghurt and bio-yoghurts made of goat’s milk. Zywnosc Nauka Technol. Jakosc.

[66] Costa M.P., Balthazar C.F., Rodrigues B.L., Lazaro C.A., Silva A.C., Cruz A.G., Conte Junior C.A. (2015). Determination of biogenic amines by high-performance liquidchromatography (HPLC-DAD) in probiotic cow’s and goat’s fermented milks and acceptance. Food Sci. Nutr..

[67] Widodo W., Taufiq T.T., Anindita N.S. (2013). Fermented goat milk and cow milk produced by different starters of lactic acid bacteria: Quality studies. J. Agric. Sci. Technol. A.

[68] Donato L., Guyomarc’h F. (2013). Formation and Properties of the Whey Protein/Kappa-Casein Complexes in Heated Skim Milk—A Review. Dairy Sci. Technol..

[69] Siemianowski K., Bohdziewicz K., Szpendowski J., Kołakowski P., Żylińska J., Bardowski J. (2015). Wpływ zwiększenia zawartości suchej masy w surowcu na teksturę i mikrostrukturę twarogu kwasowego. The effect of increased dry matter content of raw material on the texture and microstructure of acid tvorog. Acta Agrophysica.

[70] Foegeding E.A., Drake M.A. (2007). Invited review: Sensory and mechanical properties of cheese texture. J. Dairy Sci..

[71] Sołowiej B., Gustaw W. (2013). Wpływ chlorku wapnia na właściwości fizykochemiczne analogów serów topionych na bazie białek mleka i tłuszczu mlecznego. Effect of calcium chloride on physicochemical properties of processed cheese analogues based on milk proteins and milk fat. Żywność Nauka Technol. Jakość.

[72] Pawlos M., Znamirowska A., Szajnar K., Kalicka D. (2016). The influence of the dose of calcium bisglycinate on physicochemical properties, sensory analysis and texture profile of kefirs during 21 days of cold storage. Acta Sci. Pol. Technol. Aliment..

[73] Ziarno M., Zaręba D. (2019). The effect of the addition of microbial transglutaminase before the fermentation process on the quality characteristics of three types of yogurt. Food Sci. Biotechnol..

[74] Singh H., Waungana A. (2001). Influence of heat treatment of milk on cheesemaking properties. Int. Dairy J..

[75] Vasbinder A.J. (2002). Casein-Whey Protein Interactions in Heated Milk. Ph.D. Thesis.

[76] Sheehan J.J., Patel A.D., Drake M.A., McSweeney P.L.H. (2009). Effect of partial or total substitution of bovine for caprine milk on the compositional, volatile, non-volatile and sensory characteristics of semi-hard cheeses. Int. Dairy J..

[77] Szwocer J., Wituszyńska B., Obrusiewicz T., Najdeker M., Januszewska H. (2001). Próby zastosowania ultrafiltracji w produkcji serków twarogowych z mleka koziego. Attempts to use ultrafiltration in the production of acid-curd cheese from goat’s milk. Postępy Tech. Przetwórstwa Spożywczego.

[78] Ratu R.N., Usturoi M.G., Avarvarei B.V. (2015). Quality of Raw Cow Milk Utilised in Cheese Processing. Sci. Pap. Anim. Sci. Ser. Lucr. Ştiinţifice Ser. Zooteh..

[79] Szajnar K., Pawlos M., Znamirowska A. (2021). The Effect of the Addition of Chokeberry Fiber on the Quality of Sheep’s Milk Fermented by *Lactobacillus rhamnosus* and *Lact. Acidophilus*. Int. J. Food Sci..

[80] Pawlos M., Znamirowska A., Kluz M., Szajnar K., Kowalczyk M. (2020). Low-lactose fermented goat milks with *Bifidobacterium animalis* ssp. lactis Bb-12. J. Microbiol. Biotechnol. Food Sci..

[81] Chen L., Zhang H., Liu Q., Pang X., Zhao X., Yang H. (2019). Sanitising efficacy of lactic acid combined with low-concentration sodium hypochlorite on *Listeria innocua* in organic broccoli sprouts. Int. J. Food Microbiol..

